# Attitudes of doctors and nurses toward patient safety within emergency departments of two Saudi Arabian hospitals

**DOI:** 10.1186/s12913-018-3542-7

**Published:** 2018-09-25

**Authors:** Naif Alzahrani, Russell Jones, Mohamed E. Abdel-Latif

**Affiliations:** 1 The Medical School, College of Health and Medicine, Australian National University, Acton, ACT Australia; 2 Emergency Services Research Group, Health Simulation Centre, School of Medical and Health Sciences, Edith Cowan University, Joondalup, WA Australia; 3 Department of Neonatology, Centenary Hospital for Women and Children, Canberra Hospital, Garran, ACT Australia

**Keywords:** Patient safety, Safety attitudes, Patient safety climate, Quality improvement, Team-work

## Abstract

**Background:**

A hospital culture that promotes and insures patient safety is a critical aspect for the effective delivery of hospital services and patient care. Yet there are significant patient health and safety issues in hospitals worldwide. This study aims to investigate doctors’ and nurses’ attitudes toward patient safety in the emergency departments (ED) of two Saudi hospitals.

**Method:**

A cross-sectional survey using a validated Safety Attitudes Questionnaire (SAQ) was used. Total of 503 ED doctors and nurses completed SAQ. Correlation analysis, using Spearman’s Rho, was performed between the number of incidents reported and each dimension of the SAQ.

**Results:**

The mean score of each SAQ dimension was < 75%, indicating that nurses and doctors generally had less than a positive safety attitudes. This was especially prominent with dimensions of stress recognition (58.1%) and perceptions of hospital management (56.9%). Furthermore, nurses reported significantly lower on the teamwork climate dimension than doctors (*p* < .01), whereas doctors reported significantly lower on the hospital work conditions dimension than nurses (*p* < .01). There was a significant negative correlation between the number of errors reported and teamwork climate, job satisfaction, and work conditions.

**Conclusion:**

Safety attitudes of doctors and nurses employed in EDs of Saudi hospitals are less than positive and correlate with the number of reported errors. Safety training interventions and management support would appear to be the most likely avenues to improve the safety attitudes and performance within Saudi ED’s.

## Background

A hospital culture that promotes and insures patient safety is a critical aspect for the effective delivery of hospital services and patient care. Yet there are significant patient health and safety issues in hospitals worldwide [1–7] which have resulted in patient deaths, prolonged hospitalizations, irreversible disabilities, and significant financial costs [8]. Like other parts of the world, hospitals within the Kingdom of Saudi Arabian (KSA) have been associated with negative patient health and healthcare outcomes [2, 4].

The extent and details of hospital error rates associated with adverse patient events in Saudi Arabia are not easily accessed. However, it is claimed there are approximately 40,000 medical error complaints filed annually in Saudi Arabia with 3455 medical malpractice cases referred to medical legal committees [9, 10]. In one study of 642 adverse events in Saudi hospitals [11], 20.4% of errors were associated with operating rooms and 18.1% of errors were associated with emergency rooms. Research has also shown high rates of medication errors in Saudi hospitals. For example, an investigation of the error rates of 78 hospitals in Saudi Arabia [12] showed the prevalence of prescribing errors in hospital inpatient units ranges between 13 and 56 per 100 medication orders. Moreover, only 30% of the hospitals had a medication safety committee and only 9% of hospitals had a medication safety officer.

Important factors likely to impact on error rates and the quality of patient care outcomes are the safety attitudes of health care providers or a hospital’s safety climate [13, 14]. There has been some recent research to investigate the safety attitudes of health professionals in Saudi Arabian hospitals. In one study by Almutairi and colleagues (2013), the perceptions of 319 nurses about the safety climate in a major Saudi hospital was investigated. These findings showed that approximately 50% of nurses generally perceived the safety climate to be unsafe, which was particularly evident amongst the attitudes of nurses from a western background. Similar negative findings were reported in two recent studies of the perceptions of nurses. The first among 649 nurses working in Saudi Ministry of Health (MOH) hospitals [15] and, the second, among nurses working in six Saudi hospital intensive care units (ICUs) [2]. In another study [16], physicians and nurses working in a broad range of clinical areas from three Saudi Armed forces hospitals reported their safety attitudes. These findings showed that less than half of the nurses and doctors had positive attitudes towards patient safety, especially on the domains of stress recognition and perceptions of management. Moreover, positive attitudes towards patient safety were generally lower amongst nurses and physicians working in emergency departments.

Despite these findings, there are several gaps in knowledge about the safety attitudes of health professionals in Saudi hospitals. To date, there has been no reported investigation of safety attitudes in emergency departments of the MOH hospitals in Saudi Arabia. This would appear to be an important issue to clarify, given the risk-profile of emergency departments [17]. It is also the case that there has been few investigations of safety attitudes in hospital settings that have a significant mix of cultural backgrounds within their medical professionals, and how these attitudes relate to error rates [18]. Moreover, there are few research studies to compare safety attitudes of nurses and doctors at the MOH hospitals in Saudi Arabia. This is an important issue to investigate because nurses and doctors have been shown in previous research to have discrepant safety attitudes [19]. Given these gaps in the literature, the aim of this study is to assess and compare the safety attitudes of Saudi and non-Saudi doctors and nurses employed in the emergency departments of two Saudi MOH hospitals and to investigate if their safety attitudes may be reflected in hospital error rates.

## Methods

### Study design and setting

This study employed a cross-sectional survey of safety attitudes among doctors and nurses employed in emergency departments of Saudi Hospitals [20]. The study was approved by Australian Capital Territory Health Research Ethics Committee (ETHLR.16.247); Australian National University Human Ethics Committee (Protocol 2017/514) and the General Directorate for Researches and Studies, Ministry of Health, Kingdom of Saudi Arabia.

The study setting was two MOH hospitals in the capital city of Saudi Arabia, Riyadh. According to the Saudi MOH [10] there are 270 hospitals in Saudi Arabia with 40,300 beds and more than 1.7 million in-patient admissions per year. There are 71,000 nurses and 27,800 physicians employed in MOH hospitals where 2270 physicians work in emergency departments. The first hospital included in this study incorporates three hospitals: a general, a maternity, and a paediatric hospital. This site also incorporates a dental centre and a kidney centre. The first hospital has a 1500 bed capacity making it one of the largest hospitals operated by the MOH in Saudi Arabia. Its emergency and outpatient departments are among the busiest in Saudi Arabia and it is known for its great diversity of human resources [21]. The second hospital included in this study is a smaller MOH hospital with a 200-bed capacity. The choice of these two hospitals provided insight into the safety attitudes of doctors and nurses within large and modest sized hospitals.

### Safety attitudes survey

Doctors and nurses working within the emergency departments were asked to complete an English version of the SAQ, including demographic information. English language is used extensively in Saudi hospitals and has been used in similar research in Saudi Arabia [2]. Participants provided demographic information including their gender, years in their specialty, and whether they were Saudi or non-Saudi. Participants were also asked to indicate “how many errors you have reported in the 12 months” as either “No Errors”, “1–5 Errors”, “6–10 Errors”, or “More than 10 Errors”. To complete this item, participants were instructed that errors may include any accident or injury to a patient, omitted treatment, medication error, errors in transmission of doctor’s order, errors in documentation, falls, failure to change a dressing, missed treatment or omission of a required intervention.

Safety attitudes were operationalised using the Safety Attitudes Questionnaire (SAQ) developed by Sexton and colleagues [22]. The SAQ consists of 36-items that measure six safety dimensions to reflect the conceptual framework of Vincent [23]. The dimensions and sample items include teamwork climate (e.g., “The physicians and nurses here work as a well-coordinated team”), safety climate (e.g., “I would feel perfectly safe being treated here as a patient”), job satisfaction (e.g., “This is a good place to work”), working conditions (e.g., “Our levels of staffing are sufficient to handle the number of patients”), stress recognition (e.g., “When my workload becomes excessive, my performance is impaired”), and perceptions of management (e.g., “Management supports my daily efforts”). Items relating to the perceptions of management were interpreted with regard to the unit management and hospital management. Each item was answered on a 5-point Likert scale where 1 = “Strongly Disagree” and 5 = “Strongly Agree”. The SAQ has demonstrated strong psychometric properties with excellent reliability and validity in terms of construct and discriminant validity [20]. Moreover, the SAQ has been found to be a valid measure of safety attitudes across different cultural contexts including the USA [7], Saudi Arabia [2], Egypt [1], and Palestine [24].

### Data analysis

For ease of interpretation and consistent with previous research [7], response scores were transformed to a 100-point scale using the following equation: (Mean dimension score – 1) × 25 = the mean score expressed as a percentage where scores of 75 and above are judged to reflect a positive attitude toward that sub-scale domain. The reliability of each dimension was tested with the Cronbach’s alpha statistic. Independent t-tests were employed to compare mean dimension scores of safety attitudes between professional groups (doctors vs. nurses), nationality (Saudi vs. non-Saudi) and hospitals (the first hospital vs. the second hospital). To control for familywise error rate due to the use of multiple t-tests, the Bonferroni correction was made, such that the significance level was set to .01. Correlation analysis was performed to determine the relationship between each dimension of the SAQ. As reporting errors was an ordinal variable, Spearman’s Rho was employed as the correlation test statistic.

## Results

### Survey results

The demographic characteristics of survey participants are presented in Table 1. A total of 503 particpants completed the survey with a higher number of participants being employed at the first hospital (89.9%) and there were more women than men respondents overall (79.1% vs 19.5%). There were also more respondents from a non-Saudi background and a higher number were employed as nurses and worked in an adult emergency department. The background of participants also varied between doctors and nurses. Whereas a much larger proportion of nurses were from a non-Saudi background, the number of Saudi and non-Saudi doctors was very similar. Responses also showed the number of years participants had been employed in their specialty was quite varied, with most employed between 3 and 10 years. Finally, most participants reported no errors in the last 12 months, however, 33.0% of participants reported between 1 and 5 errors.

**Table 1:** The sociodemographic characteristics of the study respondents

		Number (%) (*n* = 503)
Gender	Men	98 (19.5)
	Women	398 (79.1)
	Missing	7 (1.4)
Nationality	Saudi	161 (32.0)
	Non-Saudi	335 (66.6)
	Missing	7 (1.4)
Profession	Nurse	363 (72.2)
	Doctor	139 (27.6)
	Missing	1 (0.2)
Years in Specialty	< 6 months	71 (14.1)
	6–11 months	31 (6.2)
	1–2 years	97 (19.3)
	3–4 years	109 (21.7)
	5–10 years	124 (24.7)
	11–20 years	45 (8.9)
	> 21 years	15 (3.0)
	Missing	11 (2.2)
Hospital	The first	452 (89.9)
	The second	51 (10.1)
ER Department	Adult	341 (67.8)
	Paediatrics	78 (15.5)
	Maternity	84 (16.7)
Errors	None	281 (55.9)
	1 to 5	166 (33.0)
	6 to 10	23 (4.6)
	10 or more	10 (2.0)
	Missing	23 (4.6)
Saudi	Doctors	66 (13.1)
	Nurses	95 (18.9)
non-Saudi	Doctors	71 (14.1)
	Nurses	263 (52.3)
	Missing	8 (1.6)

Data are presented as number (%)

### Reliability and Intercorrelations of SAQ dimension

Means, alpha reliabilities and intercorrelations between the seven dimensions were calculated and are presented in Table 2. The data showed the mean score for all dimensions was below 75 points. A score of 75 and above indicates a positive attitude. Therefore these results indicated that nurses and doctors had less than positive attitudes toward patient safety. This was especially prominent with mean scores on stress recognition and perceptions of hospital management, which were 58.08% and 56.93%, respectively. Only job satisfaction approached the positive range. Participants also rated the hospital work conditions as less than positve, but not comparatively more negative than other dimensions. The analysis further showed that each scale demonstrated a good and comparatively high level of reliability such that no dimension could be considered to be poorly constructed. Intercorrelational data also showed the expected moderate relationships between the dimensions, except for the Stress Recognition subscale which was generally not correlated with any of the other dimensions.

**Table 2:** Means, intercorrelations and alpha reliabilities of the SAQ dimension s

	Mean	SD	JS	SR	PM-u	PM-h	SC	TC	WC
JS	72.52	21.54	**.85**						
SR	58.08	24.96	.05	**.79**					
PM-u	60.03	19.89	.42^b^	.13^a^	**.81**				
PM-h	56.93	20.51	.43^b^	.15^b^	.67^b^	**.82**			
SC	64.49	16.97	.62^b^	−.03	.43^b^	.47^b^	**.73**		
TC	66.13	18.18	.62^b^	.02	.42^b^	.41^b^	.63^b^	**.74**	
WC	63.18	22.31	.50^b^	−.01	.44^b^	.53^b^	.42^b^	.43^b^	**.79**

Alpha reliabilities are shown on the diagonal in bold type; ^a^ = *p* < .05, ^b^ = p < .01; JS denotes job satisfaction; SR, stress recognition; PM-u, perceptions of unit management; PM-h, perceptions of hospital management; SC, safety climate; TC, teamwork climate and WC, working conditions

### Between group comparisons

Analysis was conducted to compare the mean SAQ dimension score between doctors and nurses with the relevant means shown in Table 3. Independent t-tests were conducted to test for any significant difference between the mean SAQ dimensions as a function of participant’s occupation. The analysis showed nurses reported significantly lower evaluations of teamwork climate, *t*(497) = 2.85, *p* < .01, whereas doctors reported significantly lower evaluations of the hospital work conditions, *t*(497) = 2.53, *p* < .01. Although only approaching statistical significance, the findings also showed a trend for doctors to rate the safety climate dimension lower than nurses, and to rate the unit management more positively than nurses.

**Table 3:** Mean SAQ subscale score as a function of profession

	Doctors		Nurses		*t*	*p*
	*M*	*SD*	*M*	*SD*		
Teamwork Climate	69.84	20.76	64.69	16.91	2.85	.01
Safety Climate	62.28	20.97	65.35	15.12	1.80	.07
Job satisfaction	71.67	23.42	72.88	20.83	0.56	.57
Stress recognition	60.61	26.54	57.12	24.33	1.40	.16
Unit Management	62.62	19.21	59.00	20.11	1.82	.07
Hospital Management	57.61	20.73	56.64	20.46	0.46	.64
Work conditions	59.09	25.88	64.72	20.62	2.53	.01

Mean comparisons were also conducted to compare safety attitudes between Saudi and non-Saudi nurses and doctors overall. The results from independent t-tests displayed in Table 4 showed non-Saudi nurses and doctors reported relatively similar ratings of their hospital on the SAQ dimensions, although non-Saudis generally rated most dimensions of the SAQ lower than Saudis. However, it was only in the case of perceptions of unit management where Saudi and non-Saudi nurses and doctors significantly differed; nurses’ and doctors’ perceptions of their unit management were lower amongst non-Saudis than Saudis, *t*(497) = 2.89, *p* < .01.

**Table 4:** Mean SAQ subscale score as a function of nationality

	Saudi		Non-Saudi		*t*	*p*
	*M*	*SD*	*M*	*SD*		
Teamwork Climate	67.64	19.15	65.34	17.70	1.31	.19
Safety Climate	64.24	17.62	64.68	16.58	0.27	.79
Job satisfaction	74.14	21.03	71.76	21.73	1.15	.25
Stress recognition	57.42	26.56	58.35	24.36	0.39	.70
Unit Management	63.79	19.90	58.28	19.80	2.89	.01
Hospital Management	57.56	21.51	56.92	20.07	0.32	.75
Work conditions	64.98	22.66	62.31	22.12	1.25	.21

Between group comparisons on the mean SAQ dimensions were made with respect to the hospital employment location of nurses and doctors. As shown in Table 5, doctors and nurses from the second hospital rated their job satisfaction and work conditions as significantly lower than nurses and doctors from the first hospital site, *t*(497) = 2.64, *p* < .01 and *t*(497) = 5.04, *p* < .01, respectively. Indeed, the difference in rating of work conditions was quite marked, with doctors and nurses from the second hospital showing quite poor ratings of their hospital working conditions.

**Table 5:** Mean SAQ subscale score as a function of hospital location

	First hospital		Second hospital		*t*	*p*
	*M*	*SD*	*M*	*SD*		
Teamwork Climate	66.20	18.22	65.47	17.94	0.27	.79
Safety Climate	64.80	16.54	61.50	20.73	1.26	.21
Job satisfaction	73.38	20.97	65.02	25.05	2.64	.01
Stress recognition	57.51	25.00	63.13	24.23	1.51	.13
Unit Management	60.36	20.28	57.13	16.02	1.10	.27
Hospital Management	57.50	20.72	51.96	17.95	1.79	.07
Work conditions	64.80	21.57	48.30	23.65	5.04	.01

In the final analysis reported, correlational analysis was performed between the number of incidents reported and each dimension of the SAQ with the results displayed in Table 6. These findings showed a significant negative correlation between the number of errors reported and teamwork climate, job satisfaction, and work conditions.

**Table 6:** Correlation between safety attitudes and the number of reported errors

	Errors	
	*rho*	*p*
Teamwork Climate	−.13	.00
Safety Climate	−.04	.35
Job satisfaction	−.10	.03
Stress recognition	.01	.88
Unit Management	−.02	.72
Hospital Management	.04	.42
Work conditions	−.11	.02

## Discussion

The aim of this study was to investigate safety attitudes of doctors and nurses employed in emergency departments of two MOH hospitals in Saudi Arabia. A large sample of participants (*n* = 503) completed the SAQ to measure their safety attitudes and the number of medical errors they had observed in the previous year. Overall, the findings showed nurses and doctors have less than positive attitudes toward patient safety on each dimension of the SAQ. This was especially the case with perceptions of hospital management and stress recognition. Nevertheless, participants in this study showed comparatively higher and more positive scores on job satisfaction. These findings are consistent with previous Saudi research on safety attitudes of hospital staff [4, 15] and mirror findings that physicians and nurses working in hospital ICUs had less than positive safety attitudes on the domains of stress recognition and perceptions of management [16].

Findings also showed some significant differences between the safety attitudes of nurses and doctors. One set of results showed nurses reported lower ratings of teamwork climate and unit management than doctors. In a related finding, Thomas and colleagues [17] reported nurses rated the quality of collaboration and communication with physicians to be lower than doctors. As surmised by Thomas, these findings are likely to be associated with differences in status/authority between nurses and doctors, differential responsibilities and training, gender issues, and nursing and doctor cultures. The findings from this study may reflect such issues given Saudi Arabia is stronger on the acceptance of status differences between professions and genders than Western nations [18]. Interestingly, the findings showed doctors reported lower evaluations of the hospital work conditions and the safety climate than nurses. Together, these findings suggest the safety issues associated with the physical work environment are a focus for doctors in this study, whereas the safety issues associated with the human resource components of the hospital are a focus for nurses.

Like other research findings [4, 15, 16], non-Saudi doctors and nurses generally reported lower evaluations of the safety culture than Saudi doctors and nurses; this was especially the case with the perceptions of their unit management. It is likely that cultural differences in safety attitudes are a product of divergent values, traditions, beliefs, behaviours, language and even of the management and leadership style. From a cross-cultural perspective [18], non-Saudis working in Saudi hospitals may be more likely to express negative attitudes about safety culture because they are more open to expressing their individual views and less likely to be concerned with questioning the hospital authority than Saudis. The findings also showed doctors and nurses working at the second hospital reported lower safety attitudes across most dimensions than those working at the first hospital. Indeed, staff at the second hospital had very low ratings of the quality of work conditions and hospital management and reported low job satisfaction.

Participants in this study provided some details about the number of medical errors they have reported in the last year with 39.6% indicating they had reported at least one error. Moreover, correlational analysis showed a significant negative correlation between the number of errors reported and teamwork climate, job satisfaction, and work conditions. Although the assumed relationship between safety attitudes and hospital error rates has not been clearly and unequivocally shown in the research literature [25, 26], hospital error rates have been considered by staff to reflect long work hours, high patient numbers, a lack of communication and poor management support [9]. As such, the findings of this study provides some indication that more positive safety attitudes are associated with fewer reported errors.

Altogether, the findings contribute to the literature by being one of the first studies to report safety attitudes in emergency departments of MOH hospitals in Saudi Arabia and by showing that the safety attitudes of doctors and nurses are less than positive. The findings also showed that doctors and nurses in emergency departments hold different safety attitudes that maybe due in part to their differential concern for safety issues associated with the work environment and human resources, respectively. There were also differences in the safety attitudes of Saudi and non-Saudi medical staff that have been reported elsewhere in the literature [4, 15, 16], which may reflect differences in cross-cultural values. Finally, the findings provide some confirmation to the reasoning that more positive safety attitudes would be reflected in a lower number of reported errors within emergency departments of hospitals.

The findings that doctors and nurses in Saudi emergency departments report less than positive safety attitudes suggest that interventions to enhance patient safety may need to focus on improving the safety culture of hospitals. Indeed, research has shown that safety attitudes can be positively affected by safety training interventions and other methods to highlight the importance of patient safety culture in hospitals [27–29]. Direct training of employees and ‘executive walk around’s with a focus on improving safety and identifying hazards and risks has also been shown to positively influence safety attitudes [30]. This latter finding appears to link to the findings of this study and other research [5, 7] wherein lack of management support has been associated with lower safety attitudes. Whereas providing resources for safety training and management support would be a challenge for smaller hospitals, such interventions are likely to improve patient outcomes and reduce hospital error rates.

Apart from the general restrictions of cross-sectional survey designs, the findings of this study are subject to several methodological limitations. Due to the sensitive nature of reporting errors, participants were only asked to provide an indication of the number of medical errors they had reported in the last year limiting generalisations about the impact of safety attitudes on error rates. The low number of reported errors in this study compared to other studies [11, 12], should be treated with caution as they may reflect participants’ unwillingness to disclose errors rather than actual low error rates. Although precise data on error rates may prove a challenge to access, future research on the relationship between safety attitudes and error rates would benefit from using validated indicators of the number, type and severity of hospital error rates. Another limitation concerns the generalizability is related to the fact that the results are based on self-reported questionnaires (in the English language) and that the study was conducted in only a few Saudi Arabian hospitals. However, as mentioned elsewhere, English language is used extensively in Saudi hospitals and has been used in similar research in Saudi Arabia. A further limitation was that participants were only asked to indicate their nationality as either Saudi or non-Saudi. More extensive insights about the effect of cultural background on safety attitudes would be gained in future research with questions that identify specific nationalities and their commensurate cultural values which are likely to affect differential safety attitudes. Despite these limitations, the study accessed a large sample of doctors and nurses and employed a well-validated and reliable scale to measure safety attitudes.

## Conclusion

This study contributes to knowledge about safety attitudes in emergency departments of Saudi hospitals in several ways. The findings show safety attitudes of doctors and nurses employed in emergency departments of Saudi hospitals are less than positive and correlate with the number of reported errors. Importantly, the findings show that nurses and doctors working in Saudi hospitals show quite low safety attitudes compared to staff in other hospital jurisdictions [22] and raise implications for how cross-cultural differences in values may impact on the effectiveness of hospital safety administration. Moreover, the professional and national background of doctors and nurses appear to differentially relate to their safety attitudes and may similarly reflect cross-cultural differences in values. Finally, the findings suggest future research in the Saudi context would focus on identifying the relationship between safety attitudes and hospital error rates more precisely, further investigate how the professional and cultural background of hospital staff impact on safety attitudes, and test how training interventions and management support may improve the safety attitudes and performance of Saudi hospitals and contribute to patient welfare.

## Abbreviations

ACT: Australian Capital Territory
ED: Emergency department
KSA: Kingdom of Saudi Arabia
MOH: Ministry of Health
SAQ: Safety Attitudes Questionnaire
